# Simultaneous treatment of parapelvic renal cysts and stones by flexible ureterorenoscopy with a novel four-step cyst localization strategy

**DOI:** 10.1590/S1677-5538.IBJU.2018.0074

**Published:** 2018

**Authors:** Ning Kang, Xing Guan, Liming Song, Xiaodong Zhang, Junhui Zhang

**Affiliations:** 1 Department of Urology, Institute of Urology, Capital Medical University, University Beijing Chaoyang Hospital

**Keywords:** Calculi, Lithotripsy, Renal Colic

## Abstract

**Objective:**

To assess the safety, feasibility, and efficacy of simultaneous treatment of parapelvic renal cysts and stones by flexible ureterorenoscopy with a novel four-step cyst localization strategy in selected patients.

**Patients and Methods:**

We retrospectively reviewed 11 consecutive cases of parapelvic renal cysts with concomitant calculi treated by flexible ureterorenoscopy and laser lithotripsy (FURSL). Marsupialization was performed subsequently with holmium: YAG laser in our institution. Fragmentation was used to manage renal stones and a novel four-step cyst localization strategy was applied in each case for marsupialization.

**Results:**

There were no intraoperative complications. Two cases of cystitis were reported postoperatively. The mean operative times of FURSL and marsupialization were 23.6 ± 3.9 minutes and 29.1 ± 9.7 minutes, respectively. During marsupialization, seven patients underwent the first two steps of the new strategy, two patients underwent three steps and two patients underwent all four steps. The mean reduction in hemoglobin level was 4.7 ± 1.7 g / L (range 3-8 g / L). The mean length of hospital stay was 1.2 ± 0.4 days. During a mean follow-up duration of 18 months, all cases remained stone-free and there was no stone recurrence. Parapelvic cysts became undetectable in eight cases and decreased in size by at least half in three cases.

**Conclusion:**

With appropriate patient selection, FURSL and marsupialization with a four-step cyst localization strategy is feasible, safe, and effective in treating parapelvic renal cysts with concomitant calculi.

## INTRODUCTION

The prevalence of renal cysts in the general population is about 5%, and their incidence increases with age (1). Most simple renal cysts form peripherally and, despite their frequency, only 8% of patients become symptomatic. Parapelvic cysts are less common but are more likely to be symptomatic. As these expand, the cystic pressure gradually exceeds the pressure of intrapelvic urine, leading to progressive obstruction (2, 3). Compression of the renal collecting system or the renal pedicular vessels may occur.

Symptomatic parapelvic cysts may present with flank pain, infection or hematuria, as well as renin-mediated hypertension caused by vascular compression (4). Stone formation is probably secondary to obstruction and infection.

Currently available endoscopic management options for parapelvic cysts include antegrade percutaneous nephroscopic ablation, retrograde flexible ureterorenoscopy and laparoscopic marsupialization by transperitoneal or retroperitoneal access (5). The retrograde approach is particularly effective and has a low complication rate. Other benefits include its minimally invasive nature and a short hospital stay after surgery (6).

Published research on endoscopic surgery for parapelvic cysts alone is plentiful, but few studies have investigated the management of parapelvic cysts with concomitant renal calculi. As far as we know, there are also no standard guidelines concerning the localization of parapelvic cysts during endoscopic surgery. We summarize four steps for localizing parapelvic cysts and describe our initial clinical experience in dealing with renal parapelvic cysts with concomitant calculi.

## MATERIALS AND METHODS

### Patients

We retrospectively reviewed 11 consecutive patients with parapelvic renal cysts with concomitant calculi treated by flexible ureterorenoscopy and laser lithotripsy (FURSL) and subsequent marsupialization with holmium: YAG laser in our institution between October 2013 and August 2016.

Inclusion criteria were as follows:

1. presence of a parapelvic cyst compressing the renal pelvis or renal calyx;
2. presence of secondary renal calculi larger than 0.5 cm in size caused by obstruction and failure of conservative therapy and extracorporeal shock wave lithotripsy (ESWL);
3. absence of a history of ureteral stricture and 4) symptoms such as flank pain or hematuria that progress despite 6 months or more of conservative therapy. Patients whose cysts were suspicious for malignancy on imaging or who exhibited severe hydronephrosis were excluded from the study.

All patients were preoperatively evaluated based on a complete blood count, routine biochemistry, coagulation parameters and radiographic imaging. Those with a positive urine culture were treated accordingly. Imaging evaluations included plain films of the kidneys, ureters and bladder (KUB), renal ultrasonography, intravenous urography (IVU) and / or contrast-enhanced spiral computed tomography (CT) to define the collecting system anatomy, the renal hilum and the location of the parapelvic cysts. The cyst sizes were measured using their longest axes. Informed consent was obtained, including for the conduct of alternative surgical procedures if necessary. A color Doppler ultrasound machine with a 3.5-MHz transducer (Hitachi Aloka, Tokyo, Japan) was employed in this study.

### Procedures

Under general anesthesia, patients were placed in the Galdakao-modified supine Valdivia (GMSV) position, which allowed both antegrade and retrograde renal access. Intravenous antibiotics were given preoperatively. The ureteral orifice was cannulated with a hydrophilic guidewire (0.035 / 0.038-inch, Cook^®^ Medical, Bloomington, IN, USA).

After confirming guidewire placement in the renal pelvis by ultrasound, the rigid ureteroscope (Richard Wolf GmbH, Germany) was used to evaluate the relevant ureter. The ureteral access sheath (12 / 14-Fr, 13 / 15-Fr, Cook^®^ Medical, Bloomington, IN, USA) was inserted into the ureteropelvic junction. Following preoperative imaging, the operator surveyed the entire renal pelvis and individual calyces sequentially using a flexible ureterorenoscope (YC-LF-A Youkang Company, China) to locate the parapelvic cysts and the renal calculi. Once the renal calculi were found, the operator tried to relocate the stone from the affected calyx to the renal pelvis using a 2.2-Fr zero-tip nitinol stone retrieval basket (Cook^®^ Medical, Bloomington, IN, USA). Afterwards, stone fragmentation was achieved with a holmium: YAG laser set at an energy level of 1-2 J and at a rate of 5-10 Hz. Stones were effectively broken down into fragments that were easy to pick up using the same basket.

The flexible ureterorenoscope was used to determine the exact position of the parapelvic cysts, and marsupialization was accomplished using a holmium laser with a 276-μm fiber. We routinely performed four steps to locate the cysts, as described below.

Step 1: Since the typical renal cyst appears transparent with black and blue areas, we initially tried to find the cyst wall by direct visualization.

**figure 1:**
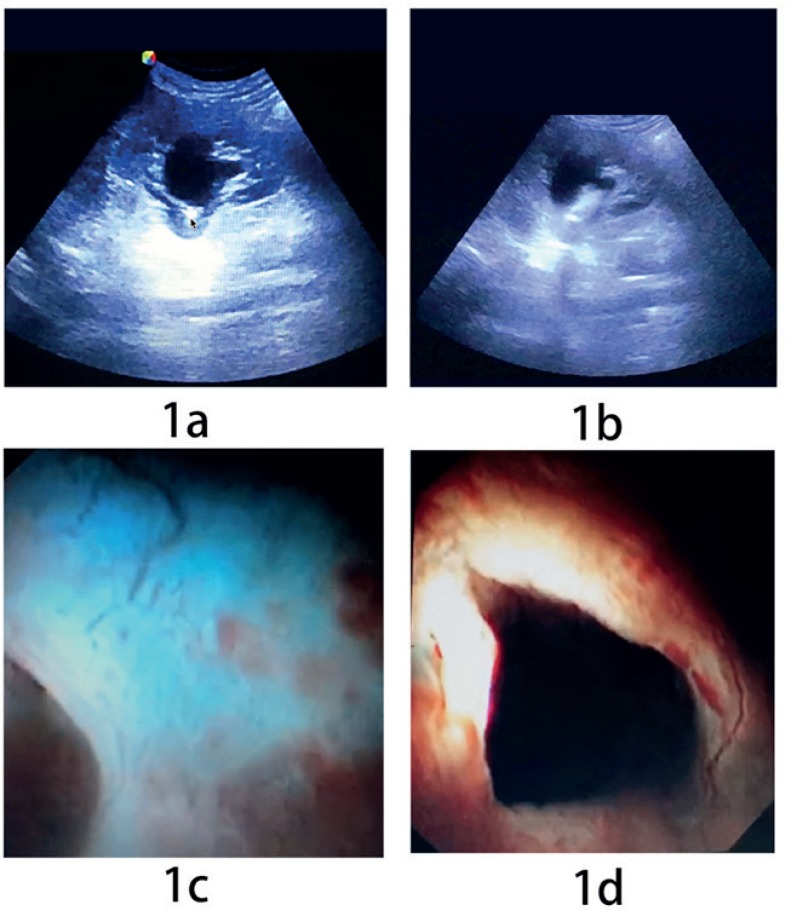
a) The flexible ureterorenoscope pushing against the cyst wall the arrow shows the tip of the flexible ureterorenoscope; 1b) Laser-assisted incision and internal drainage, with typical “smoking sign”; 1c) Under the flexible ureterorenoscope, some areas of the renal pelvis appear blue; 1d) visualization of the light from the antegrade fiber-endoscope in the darkened renal pelvis.

Step 2: The flexible ureterorenoscope was guided close to the cyst wall in real time using ultrasound, noting that the sonographic characteristic of a cyst is an anechoic area with posterior acoustic enhancement. After confirmation by the operator that the flexible ureterorenoscope was pushing against the cyst wall (Figure-1a), the laser was triggered for incision and drainage. The typical smoking sign was observed (Figure-1b).

Step 3: If the initial two steps were unsuccessful, the second surgeon performed percutaneous renal cyst puncture. Ultrasound imaging was employed to find an appropriate puncture site to avoid injuries to the bowel and other organs surrounding the kidney. Under ultrasound guidance, 10 mL of cystic fluid was extracted and the same volume of methylene blue solution (0.9% sodium chloride 1000 mL mixed with methylene blue 20-40 mg) was injected into the cyst cavity in each case to help the surgeon identify the cyst wall more accurately, using color changes detected through the flexible ureterorenoscope as a guide (Figure-1c).

**figure 2:**
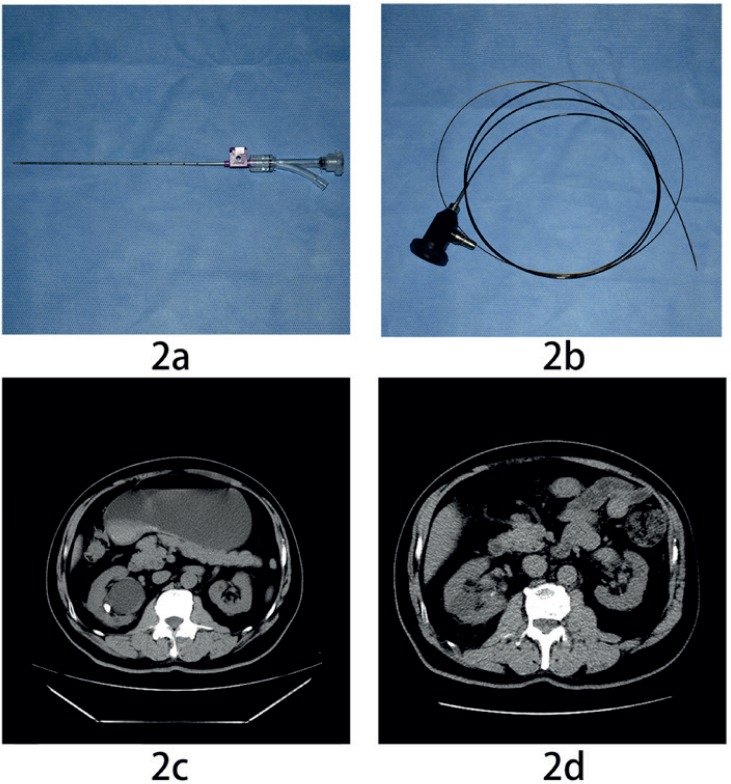
a) One adapter attached to the tip of the outer shaft; 2b) The 0.7-mm fiber-endoscope of the flexible ureterorenoscope; 2c) preoperative CT showing the right parapelvic cyst and renal stone; 2d) postoperative CT showing the absence of cysts and stones.

Step 4: If the surgeon still could not find the cyst wall accurately after step 3, a fiber endoscope was used, which consisted mainly of a 16-gauge (4.8-Fr) outer puncture shaft (Bard^®^ Magnum^®^; Tempe, AZ, USA; see Figure-2a), a 0.7-mm fiber endoscope (YC-LF-A, Youkang Company, China; see Figure-2b) and a Y-shaped three-way connector (Gateway™ Advantage Y-Adapter, Boston Scientific Corporation, USA). The adapter was attached to the tip of the outer shaft (Figure-2a), allowing the insertion of the fiber endoscope as well as syringe irrigation.

Once the outer puncture shaft was advanced into the cyst under ultrasound guidance, the fiber endoscope was inserted into the parapelvic cavity through the outer puncture shaft. The cyst's interior aspect was then completely examined. The assistant reduced the brightness of the retrograde flexible ureterorenoscope, permitting the retrograde intrarenal surgery (RIRS) operator to see the light from the antegrade fiber endoscope clearly in the darkened renal pelvis. This illuminated the cyst wall for internal drainage (Figure-1d). We set the energy level to 1.0 J and the frequency to 30 Hz to marsupialize the cyst wall.

A cruciform incision was made in an avascular area for adequate drainage. The full-thickness incision was extended to about 2-4 cm to enable communication with the collecting system. To minimize the risk of parenchymal injury especially with thick-walled cysts, a distance of 5 mm or more was maintained between the incision margin and the normal renal parenchyma. The 6-Fr double-J stent was inserted, with its proximal end inside the cyst. Two double-J stents were placed if the cyst wall was thick. The antegrade outer puncture shaft was removed immediately.

Indwelling Foley catheters were removed on postoperative day 1, and all patients were subsequently discharged from the hospital. Double-J stents were scheduled for removal 3 months after the first follow-up.

### Perioperative data

On postoperative day 1, each patient underwent renal imaging evaluation. Operative times, rates of intraoperative and postoperative complications (according to the Clavien-Dindo classification system), changes in hemoglobin, lengths of postoperative hospital stay and stone-free rates (SFRs) were also obtained for this study. All patients were followed-up 6, 12 and 24 months later for evaluation of their stone-free status and parapelvic cysts. Stone free was defined as the absence of residual stones on imaging, using enhanced CT and / or renal ultrasonography.

## RESULTS

A total of 11 patients, composed of six males and five females, underwent FURSL and marsupialization by RIRS. The mean patient age was 47.5 ± 11.9 years. The mean body mass index was 26.7 ± 3.5 kg/m^2^ (range 21.9-33.2 kg / m^2^). The mean cyst size on the preoperative CT scan was 53.6 ± 7.8 mm (range 40-65 mm). The mean stone size was 10.1 ± 1.3 mm (range 8-12 mm).

All operations were conducted successfully, with none of the cases requiring conversion to open surgery or standard percutaneous nephrolithotomy (PCNL). The mean operative times of FURSL and marsupialization were 23.6 ± 3.9 minutes and 29.1 ± 9.7 minutes, respectively. During marsupialization, seven patients underwent the initial two steps of cyst localization, two patients underwent three steps and two patients underwent all four steps. The mean reduction in hemoglobin level was 4.7 ± 1.7 g / L (range 3-8 g / L). The mean length of hospital stay was 1.2 ± 0.4 days.

There were no serious intraoperative complications such as massive hemorrhage or damage to the renal parenchyma noted. None of the patients required blood transfusion. Cystitis occurred in two patients in the postoperative period. Both were treated with oral antibiotics, and neither required read-mission.

Patients were followed up for a mean of 18 months (range 15-24 months). During follow-up, eight cysts became undetectable, while three cysts decreased in size by at least half and communicated with the renal collecting system. The SFRs obtained 6 and 12 months postoperatively were both 100% (preoperative CT Figures-2c and postoperative CT Figures-2d). There was no radiographic evidence of obstruction in any of the patients, and they were all asymptomatic.

## DISCUSSION

Although parapelvic renal cysts are a relatively rare form of renal cysts, they are adjacent to the vessels of the renal hilum and collecting system and are more frequently associated with obstruction, pain, infection and stone formation (3, 7). Therefore, parapelvic cysts more commonly require intervention.

The parapelvic cyst may expand and compress the collecting system, promote stone formation and hinder spontaneous stone passage. The initial stone sizes of five patients in our study were less than 0.5 cm; however, after long-term conservative treatment (7-12 months), the renal stones in all 11 cases enlarged rather than diminished in size. This suggests that indications for minimally invasive strategies such as endosurgery should be relaxed in cases of parapelvic cysts with concomitant calculi.

To date, there are only a few published studies describing treatments for parapelvic cysts with concomitant calculi. Chen et al. (8) reported the effectiveness of percutaneous intrarenal cyst marsupialization and simultaneous nephrolithotomy in the management of renal cysts with ipsilateral calculi in 16 patients, of which only two cases involved parapelvic cysts. In their study, the optimal puncture route was selected to marsupialize the cyst and approach the target calyx. The cyst was marsupialized into the collecting system at the dilation process. Percutaneous nephrolithotomy was performed subsequently with the 18-Fr work access. A nephrostomy tube was placed in all cases and removed 20 to 30 days later.

During the past decades, RIRS gained popularity due to significant advances in endoscope and laser technologies. The procedure often took the place of open surgery and PCNL in some complicated cases. In our study, all 11 parapelvic cysts were surrounded by thick renal parenchyma. The wide work access sites associated with PCN inevitably cause severe damage to the kidney. Intuitively, the retrograde approach may be less invasive in these patients.

To prevent stone debris from depositing in the opening of parapelvic cysts, we performed FURSL prior to marsupialization. We first attempted to extract the stone through the basket, but if the stone was too large to relocate to the renal pelvis, we divided it into smaller fragments using the fragmenting technique of laser lithotripsy, which uses high energy and low frequency. Rather than producing dust, which requires low laser energy and high frequency, we created relatively large stone fragments to facilitate easier removal by the basket, thus allowing more complete stone extraction. Consequently, it is more effective in preventing stone recurrence or deposition in the opening of the cyst cavity. Renal ultrasonography was performed to ensure the absence of residual stones in the collecting system.

Compared with simple renal parenchymal cysts, percutaneous aspiration and sclerotherapy for parapelvic cysts are associated with a high recurrence rate. Moreover, serious complications such as extravasation of sclerosing agents from the renal cyst into the retroperitoneum may lead to local inflammation and consequent ureteropelvic junction obstruction, fever, abscess formation and other issues (9–11).

With the rapid development of endoscopic instruments and the improvements in surgical techniques, the treatment of parapelvic renal cysts has evolved from open surgery to minimally invasive endoscopic modalities. Currently available modalities including antegrade percutaneous marsupialization (4), retrograde ureteroscopic marsupialization (12–14), and laparoscopic marsupialization by transperitoneal or retroperitoneal access (5, 15). When dealing with parapelvic cysts with concomitant calculi, laparoscopy has some disadvantages such as the need for multiple port sites, extensive dissection and a high risk of injury to the renal pedicle.

Several studies have clearly demonstrated that PCN produces higher complication and morbidity rates than RIRS. The complications often emerge during access creation, with rates from dilation and puncture ranging from 29% to 83% (16–18).

On the other hand, improvements in technology have leveraged on growing clinical experience to expand the indications of flexible ureterorenos-copy. In 1991, Kavoussi et al. (12) reported a single case of a large peripelvic cyst treated by ureteroscopic marsupialization, however, there were no further reports because of the technical difficulty of the procedure. In 2010, Basiri et al. reported the successful ureteroscopic treatment of parapelvic renal cysts in two cases (19).

The crucial step of marsupialization is identification of the renal cyst wall while avoiding injury to the renal parenchyma or renal vessels. To date, there are no established guidelines for the localization of parapelvic cysts in RIRS, so we summarized our experience and standard protocol for addressing this problem. We routinely follow four steps to localize parapelvic cysts in RIRS. In our center, most cases could be treated easily using the initial two steps, as we demonstrated successfully in seven patients in this study. In cases where the cyst wall is relatively thick and has the same color as other parts of the renal pelvis, it is challenging for the operator to accurately locate the parapelvic cysts and to choose the best area for incision and inner drainage. Hence, the latter two steps need to be employed.

Steps 3 and 4 are more invasive but are also more useful in extensive procedures especially for complicated renal cystic disease. It is important to rule out the presence of malignancy for complicated renal cystic diseases even though preoperative radiological imaging appears negative. The fiber endoscope was introduced into the cyst to completely inspect its interior and avoid a misdiagnosis of malignancy. This technique provides more reliable information than traditional imaging modalities such as enhanced CT or MRI as a result of direct visualization. Furthermore, our percutaneous work access was only 4.8 Fr, thereby reducing the risk of complications that are associated with standard PCN.

In our study, no massive hemorrhage or serious damage to the renal parenchyma occurred perioperatively. None of the patients required blood transfusion or conversion to open surgery. These indicate that our four-step strategy is a viable alternative approach for managing parapelvic cysts.

In two of our cases, the percutaneous puncture shaft was removed immediately without nephrostomy tube placement. The mean hospital stay was only 1.2 days, which is significantly shorter than the 4 days with PCN (8) or the 3.2 days with laparoscopy (20) reported in previous studies. This shorter convalescence can be attributed to the reduced invasiveness of our procedure.

During a mean follow-up period of 18 months (range 15-24 months), all cases remained stone-free with no renal stone recurrence. Furthermore, eight parapelvic cysts became undetectable and three cases decreased in size by at least half. All of these findings indicate that our techniques promote complete drainage of cystic fluid and prevent further compression on the kidney and collecting system. We offer an alternative method that can be used for select patients with parapelvic cysts and concomitant calculi.

Our study has significant limitations. While parapelvic cysts are relatively common, their presence in combination with renal stones is not. In this study, the number of patients who required simultaneous treatment of both lesions was also quite small. Therefore, given the difficulties of designing and conducting a randomized controlled trial in this situation, we opted to perform a retrospective study instead, which would explain the lack of a control group.
